# Superior survival in right-sided versus left-sided colon signet ring cell carcinoma

**DOI:** 10.1038/s41598-020-74926-9

**Published:** 2020-10-21

**Authors:** Zhuang Zhao, Dun-wei Wang, Na Yan, Shu Pan, Zhi-wen Li

**Affiliations:** Department of Anesthesiology, First Hospital of Jilin University, Changchun, 130021 Jilin China

**Keywords:** Cancer, Oncology, Risk factors

## Abstract

This study aimed to explore the association of tumor sidedness with the prognosis of patients with colon signet ring cell carcinoma (SRCC). Eligible patients were retrieved from the Surveillance, Epidemiology, and End Results database between 2004 and 2015. Cancer-specific survival (CSS) and overall survival (OS) were compared between patients with left-sided colon SRCC and those with right-sided lesions. A total of 2660 patients were included, among them, 1983 (74.5%) had right-sided colon SRCC. Compared to patients with left-sided colon SRCC, those who had the right-sided colon SRCC showed higher proportion of white race, female, aged ≥ 65 years, receiving total colectomy and ≥ 4 regional lymph node dissection; while had lower proportion of advanced AJCC stage. Besides, right-sided patients exhibited superior 5-year CSS (32.74% vs. 25.89%, P = 0.001) and OS (27.38% vs. 23.02%, P = 0.024) rates compared with left-sided ones. Multivariate analysis revealed that tumor sidedness was an independent prognostic factor. To be specific, patients with right-sided colon SRCC showed better CSS (HR: 0.873; 95% CI 0.777–0.981; P = 0.023) and OS (HR: 0.838; 95% CI 0.753–0.965; P = 0.002). Moreover, subgroup analysis demonstrated superior CSS and OS for right-sided patients in most subgroups. Tumor sidedness was an independent prognostic indicator for colon SRCC. Besides, patients with right-sided colon SRCC have superior prognosis than those with left-sided lesions.

## Introduction

Colorectal cancer (CRC) still ranks the second place among the all causes of cancer-related deaths in US, and it represents a severe health issue worldwide^1^. CRC can be classified intodifferent subtypes, among which, the colorectal signet ring cell carcinoma (SRCC) has aroused wide attentions recently. Laufman and Saphir first described SRCC in 1951^2^. Primary SRCC usually occurs in stomach while rarely develops in the colorectum^3–8^.

In the last few years, great attention has been paid to the differentiation between left-sided and right-sided CRC. As reported in recent research, right-sided CRC shows an increasing morbidity in the last decade^9,10^, thus prompting us to investigate the possible causes for such variation in tumor sidedness. Recent systematic review has suggested that great heterogeneities were detected between the left-sided and right-sided CRC in terms of their pathology, epidemiology, genetic mutations and clinical presentations^11^. At present, no consensus is reached on the prognosis for left-sided versus right-sided CRC, and it is still controversial whether tumor location significantly affects patient prognosis^12–15^. Nonetheless, no study has been carried out to investigate the role of tumor sidedness on the survival for patients with colon SRCC.

The Surveillance, Epidemiology and End Results (SEER) program is supported by the National Cancer Institute (NCI). This program contains research data of 18 different population-based cancer registries, which covers 30% of the US population^16^. In this regard, SEER has been recognized as an efficient database for investigating such rare cancers^17–19^. This work aimed to examine the relationship of tumor sidedness with the survival of patients with colon SRCC based on the SEER database.

## Materials and methods

### Ethics statement

SEER data were accessed after signing the SEER Research Data Agreement (reference number, 19817-Nov2018). Data were subsequently obtained in accordance with stipulated guidelines. All data used in this study were freely accessible and did not involve human subjects (identified by the Office for Human Research Protection), thus, institution review board approval was exempted.

### Study population

The SEER*State v8.3.6tool (August 8th, 2019) was utilized to select suitable subjects from 18 SEER regions between 2004 and 2015 (submitted in 2018). Subjects conforming to the following criteria were included: (1) patients with primary CRC; (2) pathologically confirmed SRCC according to the third edition of International Classification of Disease for Oncology (histology code: 8490/3). The exclusion criteria were listed as follows: (1) patients with multiple primary cancers; (2) clinically diagnosed alone, or based on death certificate or autopsy; (3) unavailable important information, such as surgical method and AJCC stage; (4) unknown tumor location; (5) with regard to tumor sidedness analysis (left-sided versus right-sided colon), rectal and appendiceal malignancies were eliminated; (6) without prognostic data. The remaining subjects were included as the SEER initial cohort.

### Covariates and study endpoint

Patient features were analyzed based on the following factors: year of diagnosis (2004–2007, 2008–2011, 2012–2015); insured status (uninsured/unknown, any medicaid/insured), age (˂ 65, ≥ 65); marital status (unmarried, married); gender (female, male); race (black, white or others); tumor sidedness (left-sided colon, right-sided colon); grade (grade I/II, grade III/IV, unknown); tumor size (≤ 5 cm, ˃5 cm, unknown); AJCC stage (stage I, II, III, IV); surgery(no surgery, local tumor excision /partial colectomy, total colectomy), lymph node dissections (LNDs) (none or biopsy, 1–3 regional lymph nodes removed, ≥ 4 regional lymph nodes removed, unknown), chemotherapy (no/unknown, yes), radiotherapy(no/unknown, yes). Specifically, the widowed or single (never married or having a domestic partner) or divorced or separated patients were classified as unmarried. Year at diagnosis was divided into 2004–2007, 2008–2011 and 2012–2015 according to prior studies^20,21^. Besides, tumor sidedness was divided into left-sided colon (including splenic flexure, descending colon, sigmoid colon and rectosigmoid cancers) and right-sided colon (including cecum, ascending colon, hepatic flexure and transverse colon)^12,15,22,23^. Age and tumor size were grouped in accordance with prior researches^24,25^. In addition, the staging of cancer is based on the 6th edition of AJCC stage system, which adapted to patients in the SEER database with from 2004 to 2015.

The endpoint of this study included cancer‐specific survival (CSS) and overall survival (OS). CSS was defined as the period from diagnosis to death attributed to colon SRCC. OS was defined as the period from diagnosis to death of any cause.

### Statistical analyses

Kaplan–Meier (K–M) method was employed to estimate the survival, followed by log-rank test for assessing the differences of CSS and OS. Notably, if variables had *P* values ≤ 0.1 in univariate analysis, they were incorporated into multivariate Cox proportional hazard analysis. Before multivariate analysis, Schoenfeld residual method was used to test the proportional risk assumption of Cox regression model, which revealed that the proportional risk assumption of this study was tenable. Similarly, Cox regression analysis was also used for stratified analysis. SPSS software (SPSS Inc., Chicago, USA, version 19.0) was used for statistical analysis, and GraphPad Prism 5 was utilized for plotting survival curves and generating forest plots. A two-sided *P* < 0.05 indicated statistical significance.

## Results

### Patient features

**Figure 1:**
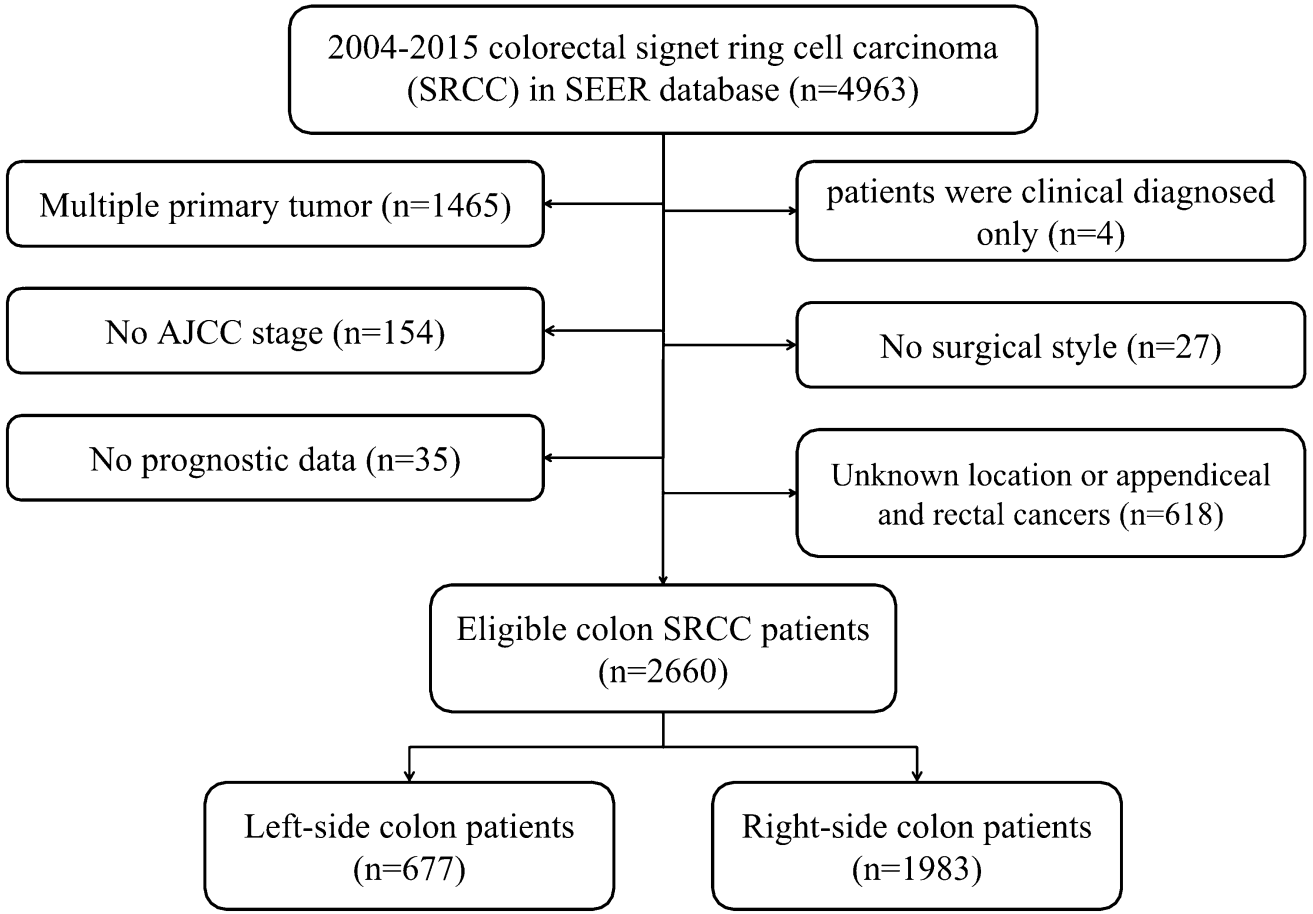
Flow chart of patient selection.

We identified 2660 eligible colon SRCC patients from the SEER database between 2004 and 2015, with a median follow-up of 16 months (range 0–155 months). Then, these subjects were classified into left-sided (n = 677, 25.5%) and right-sided (n = 1983, 74.5%) colon group. The detailed procedure of patient selection was presented in Fig. 1. Table 1 showed the baseline characteristics of included patients divided based on tumor sidedness. According to the results, differences in year at diagnosis (P = 0.049), insured status (P = 0.001), age (P < 0.001), gender (P < 0.001), race (P < 0.001), AJCC stage (P < 0.001), surgery (P < 0.001), LNDs (P < 0.001), chemotherapy (P < 0.001) and radiotherapy (P < 0.001) between two groups were statistically significant. Patients with right-sided colon SRCC had higher proportion of insured status (71.1% vs. 64.4%), aged ≥ 65 (53.7% vs. 36.9%), female (55.8% vs. 39.4%), white race (84.8% vs. 78.9%), receiving total colectomy (68.2% vs. 28.1%) and ≥ 4 LNDs (77.6% vs. 68.1%), while had lower proportion of advanced AJCC stage (79.2% vs. 86.5%), receiving chemotherapy (53.1% vs. 63.4%) and radiotherapy (2.2% vs. 10.0%) compared to patients with left-sided lesions.

**Table 1:** The clinicopathological characteristics and treatment of the included 2660 colon signet ring cell carcinoma patients.

Characteristic	Total	Right-sided colon	Left-sided colon	*P*-value
**Year at diagnosis**				0.049
2004–2007	873 (32.8%)	625 (31.5%)	248 (36.6%)	
2008–2011	863 (32.4%)	654 (33.0%)	209 (30.9%)	
2012–2015	924 (34.7%)	704 (35.5%)	220 (32.5%)	
**Insured status**				0.001
Uninsured/unknown	814 (30.6%)	573 (28.9%)	241 (35.6%)	
Any medicaid/insured	1846 (69.4%)	1410 (71.1%)	436 (64.4%)	
**Age**				< 0.001
< 65	1346 (50.6%)	919 (46.3%)	427 (63.1%)	
≥ 65	1314 (49.4%)	1064 (53.7%)	250 (36.9%)	
**Marital status**				0.283
Unmarried	1222 (45.9%)	923 (46.5%)	299 (44.2%)	
Married	1438 (54.1%)	1060 (53.5%)	378 (55.8%)	
**Gender**				< 0.001
Female	1374 (51.7%)	1107 (55.8%)	267 (39.4%)	
Male	1286 (48.3%)	876 (44.2%)	410 (60.6%)	
**Race**				< 0.001
Black	248 (9.3%)	174 (8.8%)	74 (10.9%)	
White	2215 (83.3%)	1681 (84.8%)	534 (78.9%)	
Other	197 (7.4%)	128 (6.5%)	69 (10.2%)	
**Grade**				0.903
Grade I/II	153 (5.8%)	112 (5.6%)	41 (6.1%)	
Grade III/IV	2078 (78.1%)	1549 (78.1%)	529 (78.1%)	
Unknown	429 (16.1%)	322 (16.2%)	107 (15.8%)	
**Tumor size**				0.131
≤ 5 cm	1016 (38.2%)	772 (38.9%)	244 (36.0%)	
> 5 cm	1092 (41.1%)	817 (41.2%)	275 (40.6%)	
Unknown	552 (20.8%)	394 (19.9%)	158 (23.3%)	
**AJCC stage**				< 0.001
I	108 (4.1%)	75 (3.8%)	33 (4.9%)	
II	395 (14.8%)	337 (17.0%)	58 (8.6%)	
III	976 (36.7%)	720 (36.3%)	256 (37.8%)	
IV	1181 (44.4%)	851 (42.9%)	330 (48.7%)	
**Surgery**				< 0.001
No surgery	422 (15.9%)	272 (13.7%)	150 (22.2%)	
Local tumor excision/partial colectomy	696 (26.2%)	359 (18.1%)	337 (49.8%)	
Total colectomy	1542 (58.0%)	1352 (68.2%)	190 (28.1%)	
**Lymph node dissection**				< 0.001
None or biopsy	587 (22.1%)	398 (20.1%)	189 (27.9%)	
1—3	73 (2.7%)	46 (2.3%)	27 (4.0%)	
≥ 4	2000 (75.2%)	1539 (77.6%)	461 (68.1%)	
**Chemotherapy**				< 0.001
No/unknown	1179 (44.3%)	931 (46.9%)	248 (36.6%)	
Yes	1481 (55.7%)	1052 (53.1%)	429 (63.4%)	
**Radiotherapy**				< 0.001
No/unknown	2549 (95.8%)	1940 (97.8%)	609 (90.0%)	
Yes	111 (4.2%)	43 (2.2%)	68 (10.0%)	

### Prognostic effect of tumor sidedness on patient

**Figure 2:**
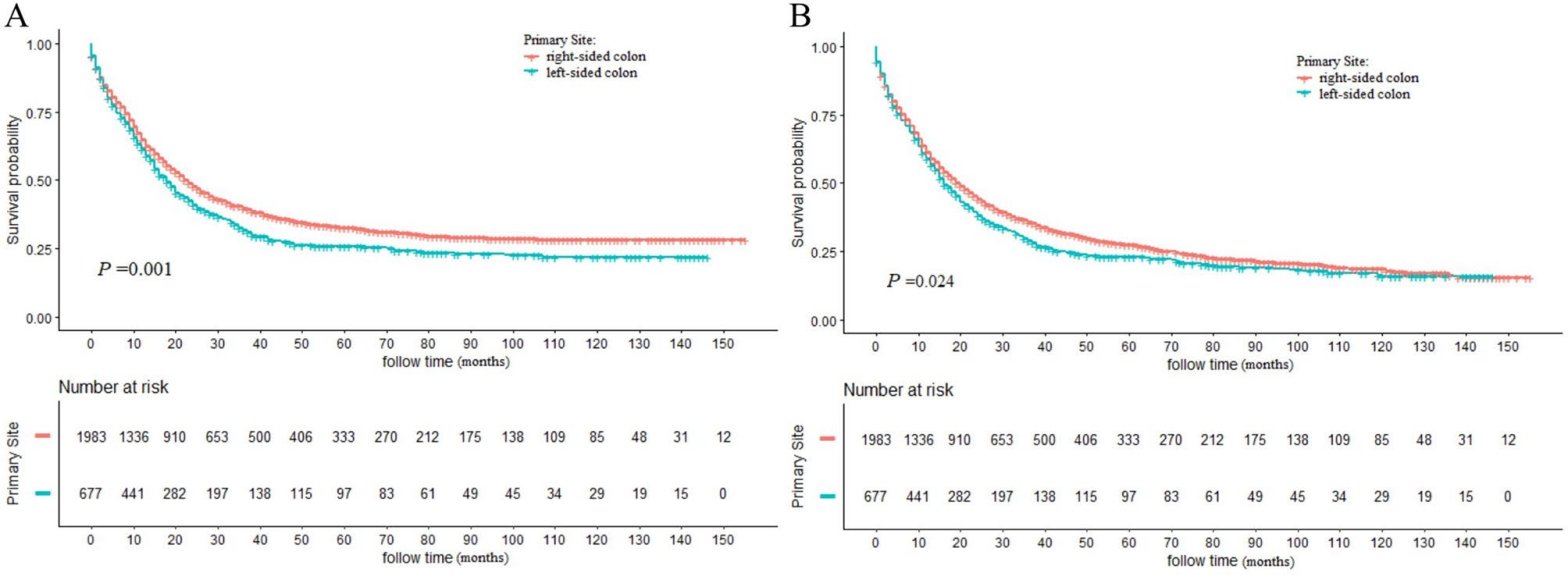
Kaplan–Meier (K–M) curves for cancer-specific survival (CSS) (**a**) and overall survival (OS) (**b**) between left-sided and right-sided SRCC patients.

Differences in CSS (*P* = 0.001) and OS (*P* = 0.024) between right-sided and left-sided groups were statistically significant (Fig. 2). The 3-, 5- and 10-year CSS rates were 31.70%, 25.89% and 21.81% in left-sided patients, which were 39.94%, 32.74% and 28.26% in right-sided patients. Meanwhile, the 3-, 5- and 10-year OS were 28.84%, 23.02% and 15.78% for left-sided SRCC patients, which were 35.89%, 27.38% and 18.43% for right-sided patients. Univariate log-rank test identified several factors related to CSS (*P* ≤ 0.1), including tumor sidedness, gender, marital status, grade, tumor size, AJCC stage, surgery, LNDs, chemotherapy and radiotherapy. After adjusting for the above factors, tumor sidedness remained as an independent risk factor of CSS. Additionally, patients with right-sided colon had significantly better CSS than left-sided ones [hazard ratio (HR):0.873; 95% confidence interval (CI) 0.777–0.981; *P* = 0.023]. Meanwhile, all aforementioned covariates and age were significantly associated with OS. Multivariate analysis showed that patients with right-sided colon also had a favorable prognosis in terms of OS (HR: 0.838; 95% CI 0.753–0.965; *P* = 0.002). The detailed results of univariate and multivariate analysis were shown in Table 2.

**Table 2:** Univariate and multivariate Cox regression analyses of cancer special survival (CSS) and overall survival (OS) for patients with colon SRCC.

Variables	CSS			OS		
	Univariate analysis	Multivariate analysis		Univariate analysis	Multivariate analysis	
	*P*-value	HR (95%CI)	*P*-value	*P*-value	HR(95%CI)	*P*-value
**Year at diagnosis**	0.488		NI	0.508		NI
2004–2007						
2008–2011						
2012–2015						
**Insured status**	0.271		NI	0.241		NI
Uninsured/unknown						
Any medicaid/insured						
**Age**	0.614		NI	< 0.001		< 0.001
< 65					Reference	
≥ 65					1.478 (1.340,1.629)	
**Gender**	0.090		0.039	0.326		NI
Female		Reference				
Male		1.110 (1.005,1.226)				
**Race**	0.221		NI	0.400		NI
Black						
White						
Other						
**Marital status**	0.025		0.013	0.001		0.017
Unmarried		Reference			Reference	
Married		0.882 (0.799,0.974)			0.896 (0.818,0.981)	
**Grade**	< 0.001		< 0.001	< 0.001		< 0.001
Grade I/II		Reference			Reference	
Grade III/IV		1.598 (1.262,2.023)	< 0.001		1.540 (1.247,1.902)	< 0.001
Unknown		1.743 (1.345,2.260)	< 0.001		1.665 (1.316,2.106)	< 0.001
**Tumor size**	< 0.001		0.001	< 0.001		0.004
≤ 5 cm		Reference			Reference	
> 5 cm		1.208 (1.077,1.354)	0.001		1.156 (1.041,1.283)	0.007
Unknown		1.231 (1.058,1.431)	0.007		1.221 (1.059,1.410)	0.006
**AJCC stage**	< 0.001		< 0.001	< 0.001		< 0.001
I		Reference			Reference	
II		1.608 (0.965,2.679)	0.068		1.628 (1.128,2.349)	0.009
III		6.919 (4.299,11.138)	< 0.001		4.629 (3.270,6.551)	< 0.001
IV		17.619 (10.944,28.366)	< 0.001		11.391 (8.034,16.149)	< 0.001
**Surgery**	< 0.001		< 0.001	< 0.001		< 0.001
No surgery		Reference			Reference	
Local tumor excision/partial colectomy		0.596 (0.482,0.738)	< 0.001		0.626 (0.511,0.766)	< 0.001
Total colectomy		0.685 (0.550,0.854)	0.001		0.727 (0.590,0.896)	0.003
**Dissected lymph node**	< 0.001		0.446	< 0.001		0.133
None or biopsy		Reference			Reference	
1—3		0.981 (0.713,1.350)	0.908		0.979 (0.727,1.317)	0.886
≥ 4		0.888 (0.729,1.083)	0.241		0.842 (0.699,1.013)	0.069
**Chemotherapy**	0.069		< 0.001	0.001		< 0.001
No/unknown		Reference			Reference	
Yes		0.528 (0.476,0,587)			0.528 (0.477,0.584)	
**Radiotherapy**	0.002		0.052	0.012		0.030
No/unknown		Reference			Reference	
Yes		1.239 (0.998,1.539)			1.261 (1.023,1.554)	
**Tumor sidedness**	0.001		0.023	0.024		0.002
Left-sided colon		Reference			Reference	
Right-sided colon		0.873 (0.777,0.981)			0.838 (0.753,0.965)	

CSS, cancer‐specific survival; OS, overall survival; NI, not included in the multivariate survival analysis; HR, Hazard ratios; CI, confidence intervals.

### Subgroup analysis of the effect of cancer sidedness on CSS and OS

**Figure 3:**
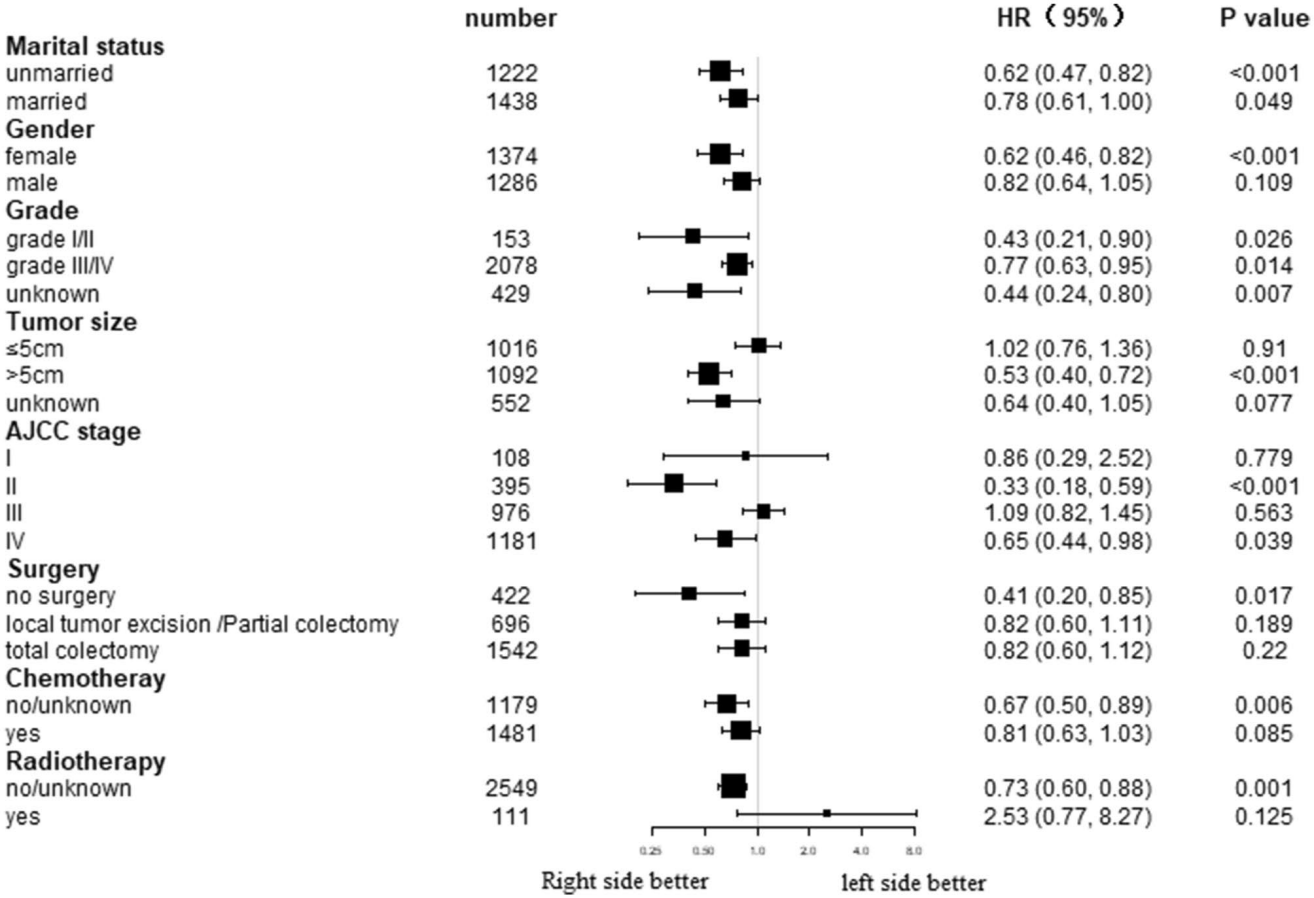
Forest plot of subgroup analysis for cancer-specific survival (CSS).

**Figure 4:**
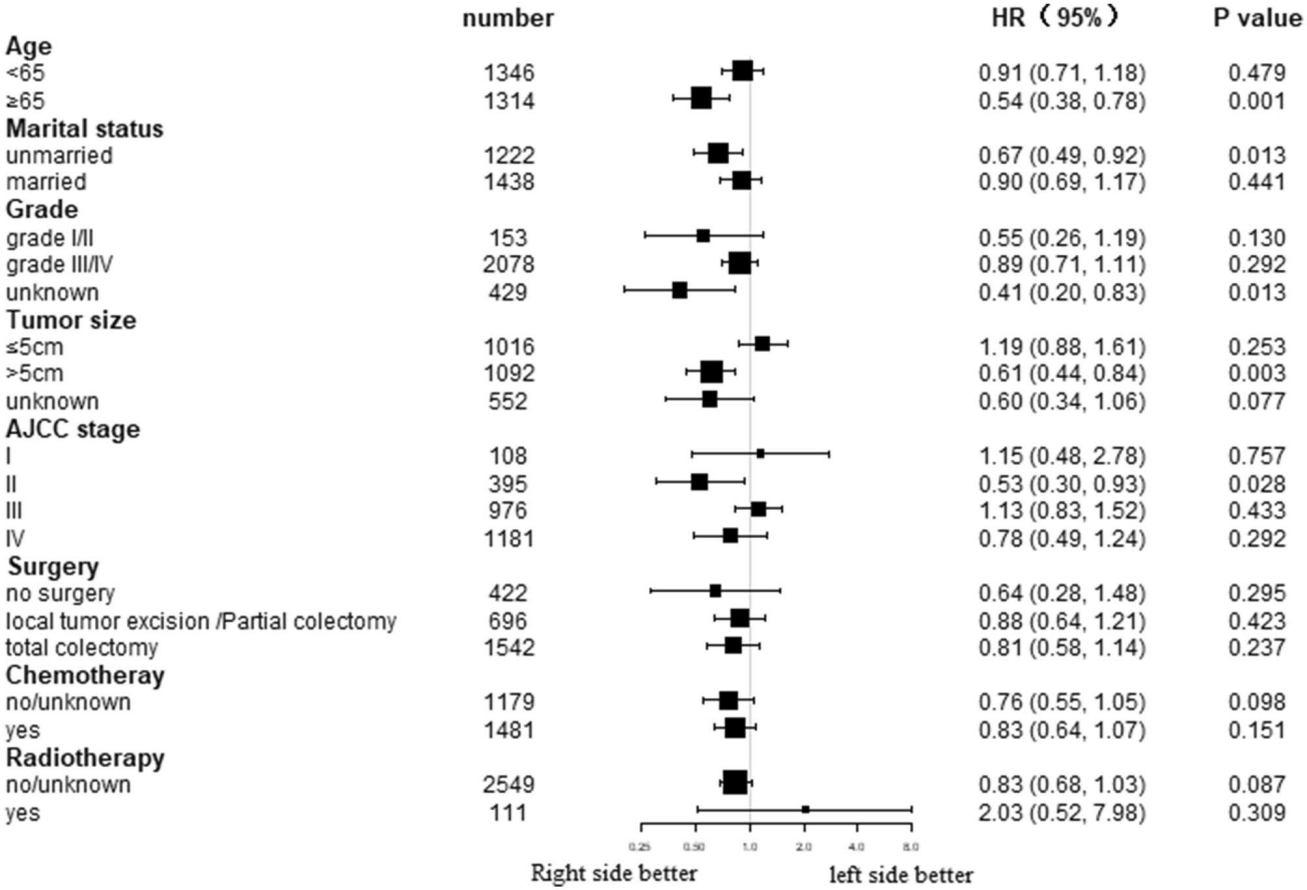
Forest plot of subgroup analysis for overall survival (OS).

The impacts of tumor sidedness on patient CSS and OS were determined based on subgroup analysis. As indicated by forest plots for subgroup analysis, right-sided patients had superior OS and CSS than left-sided patients in most subgroups (Figs. 3 and 4).

## Discussion

To our knowledge, this is the first study to explore the effect of tumor sidedness on the survival of colon SRCC patients. In order to make our data more convincing, patients were strictly screened. First of all, only pathologically confirmed primary colon SRCC patients were selected. Then, patients with secondary primary tumor were deleted. Finally, we also deleted patients with missing important clinicopathological variables, such as AJCC stage, location, etc. Finally, a total of 2660 colon SRCC patients were enrolled into analysis. And our results indicated that right-sided patients were associated with better survival and prognosis. After adjusting for tumor and demographic features, the cancer-specific death and overall death in patients with right-sided colon SRCC were reduced by 12.7% and 16.2%, respectively, relative to those in left-sided patients. In general, tumor sidedness serves as an independent factor to predict prognosis among colon SRCC patients.

As stated in studies, right-sided CRC patients are associated with older age, female, greater tumor size, more advanced tumor stages, and poor tumor differentiation^10,26–28^. These are partly consistent with our findings, except that we found that right-sided colon SRCC tended to have earlier stage.

At present, no uniform results have been obtained for the prognosis of left-sided versus right-sided CRC, and more studies revealed poorer survival in right-sided primary tumor^29–31^. For instance, Petrelli et al. performed a systematic review and meta-analysis by recruiting 66 studies, and further suggested that left-sided CRC was correlated with the decreased mortality rate (HR: 0.82; 95% CI 0.79–0.84)^29^. In addition, by enrolling stage III CRC patients (*n* = 1,869), Taieb et al. discovered that right-sided cancer patients showed worse OS (HR: 1.25; 95% CI 1.02–1.54) compared left-sided tumor patients^30^. Another study revealed the association of left-sided tumor with better survival in patients with wild-type RAS metastatic CRC^31^.

However, different outcomes have also been suggested by other studies. By adopting propensity score matching, Rene Warschkow demonstrated that right-sided CRC patients had superior OS (HR = 0.92, 95% CI 0.8–0.94, *P* < 0.001) together with better CSS (HR = 0.90, 95% CI 0.87–0.93, *P* < 0.001)^32^. Generally speaking, no consensus has been reached about the prognosis for left-sided vs. right-sided CRC. In consideration of the numerous heterogeneities (such asinclusion or exclusion criteria, study design), it remains difficult to compare those available studies.

Notably, the SEER cancer registry is beneficial because it allows us to evaluate rare cancers in a great cross-section and offers the long-term follow-up data with no intrinsic institutional bias. Nonetheless, certain limitations should be noted in our study. Firstly, 2303 (46%) patients were excluded because of the strict screening criteria in this study, which may lead to certain selection biases, but it is inevitable in retrospective studies^17,19^. Secondly, not all required data are contained in the SEER database to comprehensively answer our questions. For instance, there is no available information regarding family history, performance status, extramural vascular invasion or obstruction/occlusion status. Thirdly, the variables of chemotherapy and radiotherapy showed low sensitivity. Only a part of patients definitely received radiotherapy or chemotherapy, which is defined as "yes"; while others are uncertain, which is defined as "no/unknown". Therefore, interpreting this part of data should be extremely careful. Finally, several important prognostic information is unavailable from the SEER database, such as Microsatellite stability/Microsatellite instability (MSS/MSI) status. Although it is better to obtain more details, we believed that the present available data from SEER database could fit our research objectives very well. The above concerns should be investigated in future studies.

## Conclusions

In conclusion, our present findings suggest that tumor sidedness could serve as an independent factor to predict prognosis in patients with colon SRCC. Compared with patients with left-sided colon SRCC, the right-sided patients have superior prognosis. More in-depth research is warranted to further examine tumor heterogeneity together with the related biological factors. In addition, tumor sidedness may be identified as an independent factor when selecting the therapeutic regimen in colon SRCC management. Collaborations are valuable for merging data derived from multiple institutions in the future.
